# Exploration of the molecular mechanism of intercellular communication in paediatric neuroblastoma by single-cell sequencing

**DOI:** 10.1038/s41598-023-47796-0

**Published:** 2023-11-21

**Authors:** Jing Chu

**Affiliations:** Department of Pathology, Anhui Provincial Children’s Hospital, 39 Wangjiang East Road, Hefei, 230051 Anhui China

**Keywords:** Computational biology and bioinformatics, Genetics, Biomarkers, Molecular medicine, Oncology, Pathogenesis

## Abstract

Neuroblastoma (NB) is an embryonic tumour that originates in the sympathetic nervous system and occurs most often in infants and children under 2 years of age. Moreover, it is the most common extracranial solid tumour in children. Increasing studies suggest that intercellular communication within the tumour microenvironment is closely related to tumour development. This study aimed to construct a prognosis-related intercellular communication-associated genes model by single-cell sequencing and transcriptome sequencing to predict the prognosis of patients with NB for precise management. Single-cell data from patients with NB were downloaded from the gene expression omnibus database for comprehensive analysis. Furthermore, prognosis-related genes were screened in the TARGET database based on epithelial cell marker genes through a combination of Cox regression and Lasso regression analyses, using GSE62564 and GSE85047 for external validation. The patients’ risk scores were calculated, followed by immune infiltration analysis, drug sensitivity analysis, and enrichment analysis of risk scores, which were conducted for the prognostic model. I used the Lasso regression feature selection algorithm to screen characteristic genes in NB and developed a 21-gene prognostic model. The risk scores were highly correlated with multiple immune cells and common anti-tumour drugs. Furthermore, the risk score was identified as an independent prognostic factor for NB. In this study, I constructed and validated a prognostic signature based on epithelial marker genes, which may provide useful information on the development and prognosis of NB.

## Introduction

Neuroblastoma (NB) is the most common paediatric solid tumour located extracranially, accounting for up to 8% of paediatric malignancies^1^. This malignant tumour manifests anywhere along the sympathetic nervous system and is most often located in the abdomen along the sympathetic chain and the adrenal gland medullary region^2^. More than half of the affected patients are under 2 years of age at the time of diagnosis. The clinical course of NB is highly heterogeneous, including everything from spontaneous regression or differentiation to treatment-refractory progression despite intensive therapy. The survival rate of high-risk NB is less than 40% despite multimodal therapy, including surgery, highly intensive chemotherapy, radiation therapy, and immunotherapy^3^. Therefore, it is crucial to identify a novel gene signature for the prognosis of patients with NB and to explore novel therapeutic targets for NB.

Intercellular communication, also known as cell–cell interaction, is an essential feature of multicellular organisms. Dynamic communication networks, formed through communication and cooperation between cells, play crucial roles in numerous biological processes^4^. One of the most important forms of intercellular communication are the ligand-receptor interactions (LRIs). The ligand can either be secreted and bind to the receptor in a soluble form, or be membrane-bound and require physical proximity to the two interacting cell types^5^. The tumor microenvironment (TME) contains many cell types, including malignant, stromal, and immune cells. The identification of communication between cancer cells, and between cancer and normal cells via LRIs in the TME, helps us to understand the mechanisms of tumorigenesis, tumor progression, therapy resistance, immune infiltration, and inflammation^6^. Given the importance of LRIs in the treatment and clinical prognosis of patients with malignant tumors, therapies targeting intercellular communication have become valuable tools in clinical practice. For example, immune checkpoint inhibitors, such as ipilimumab, target CD28 or CTLA4, while pembrolizumab and nivolumab target PD1 or PDL1^6^. In recent years, the combined use of dinutuximab and immune modulators (granulocyte–macrophage colony-stimulating factor and interleukin-2) has been introduced in high-risk NB maintenance therapy^7^. Dinutuximab (ch14.18) is a chimeric monoclonal antibody targeting GD2, which is widely expressed on tumor cells derived from neuroectodermal origins, including NBs. Although anti-GD2 therapy has shown some success in the clinical treatment of NB, more than 40% of patients with NB do not respond to this targeted therapy, and some experience severe, uncontrollable neuropathic pain as a major side effect. Furthermore, while anti-GD2 immunotherapy is highly effective against small residual lesions, its efficacy against primary solid tumors is limited^7^. This limitation may be due to the complex network of cell–cell interactions in the TME and our incomplete understanding of this network. To better provide individualized treatment for patients with malignant tumors and identify suitable and effective treatment targets, we need a more comprehensive understanding of the spectrum of cell–cell interactions that occur in the TME and how these interactions impact the tumor development process and patient outcomes.

Data obtained from single-cell RNA sequencing (scRNA-seq) technology provide strong support for the analysis of human tumor heterogeneity and different subpopulations, and has proven to be key to elucidating tumor development and progression mechanisms^8^. With the maturation of single-cell isolation techniques in the TME, the availability of high-quality scRNA-seq data, and new computational models for bioinformatics analyses, a deeper exploration of the complexity of the NB microenvironment and intercellular communication has become possible. However, the prognostic value of intercellular communication-associated genes (ICAGs) in children with NB has not been evaluated. This study evaluated these ICAGs and prognosis in NB, and a prognostic model was constructed. I believe that my findings will provide information on the prognostic value of genes related to intercellular communication and preliminarily uncover the complex biological functions and immunoregulatory effects of these genes and their regulatory networks.

## Results

### Pre-processing of single-cell expression profile data and Subtype clustering analysis

**Figure 1:**
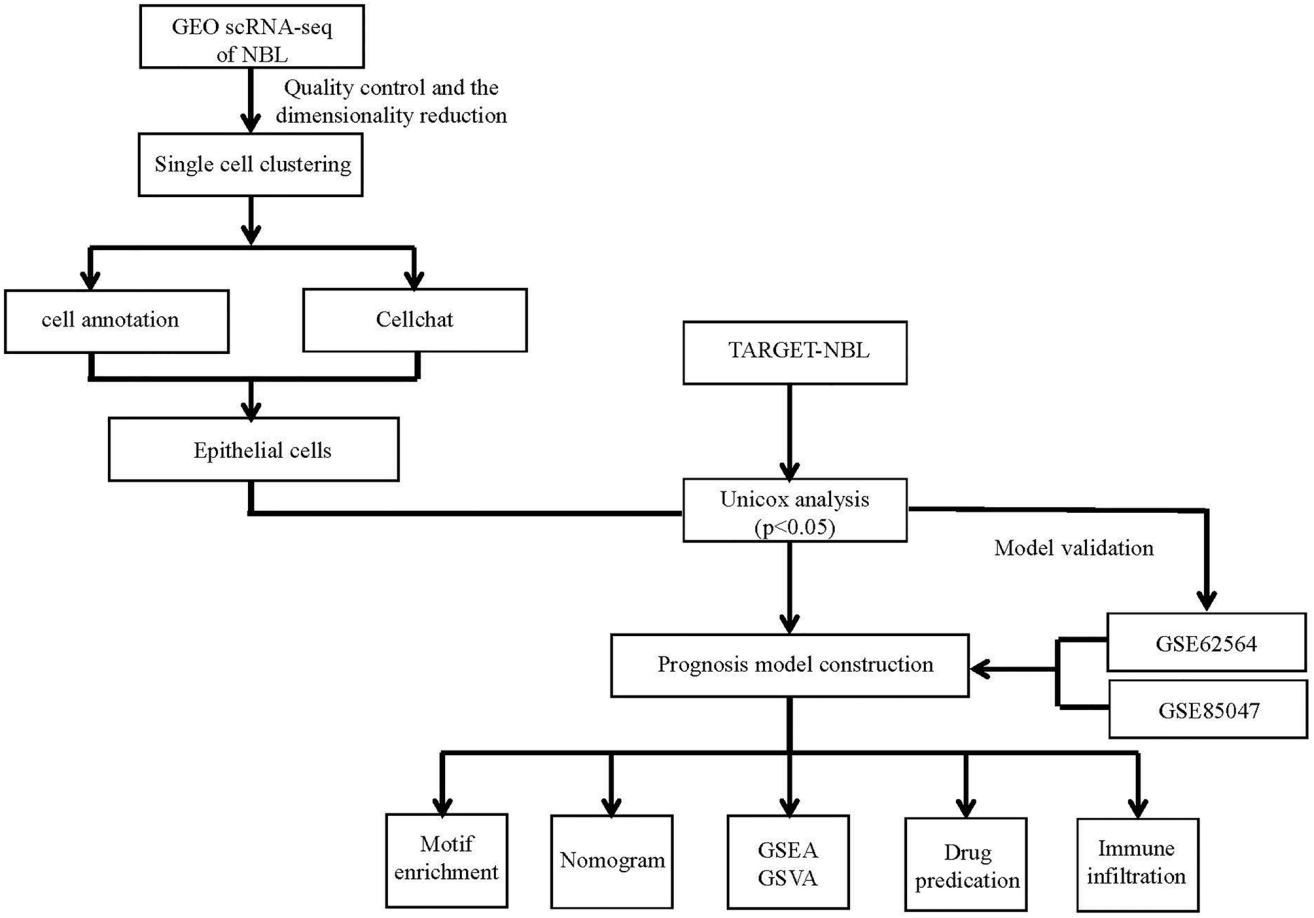
The flow chart describes the research idea and content of this study.

**Figure 2:**
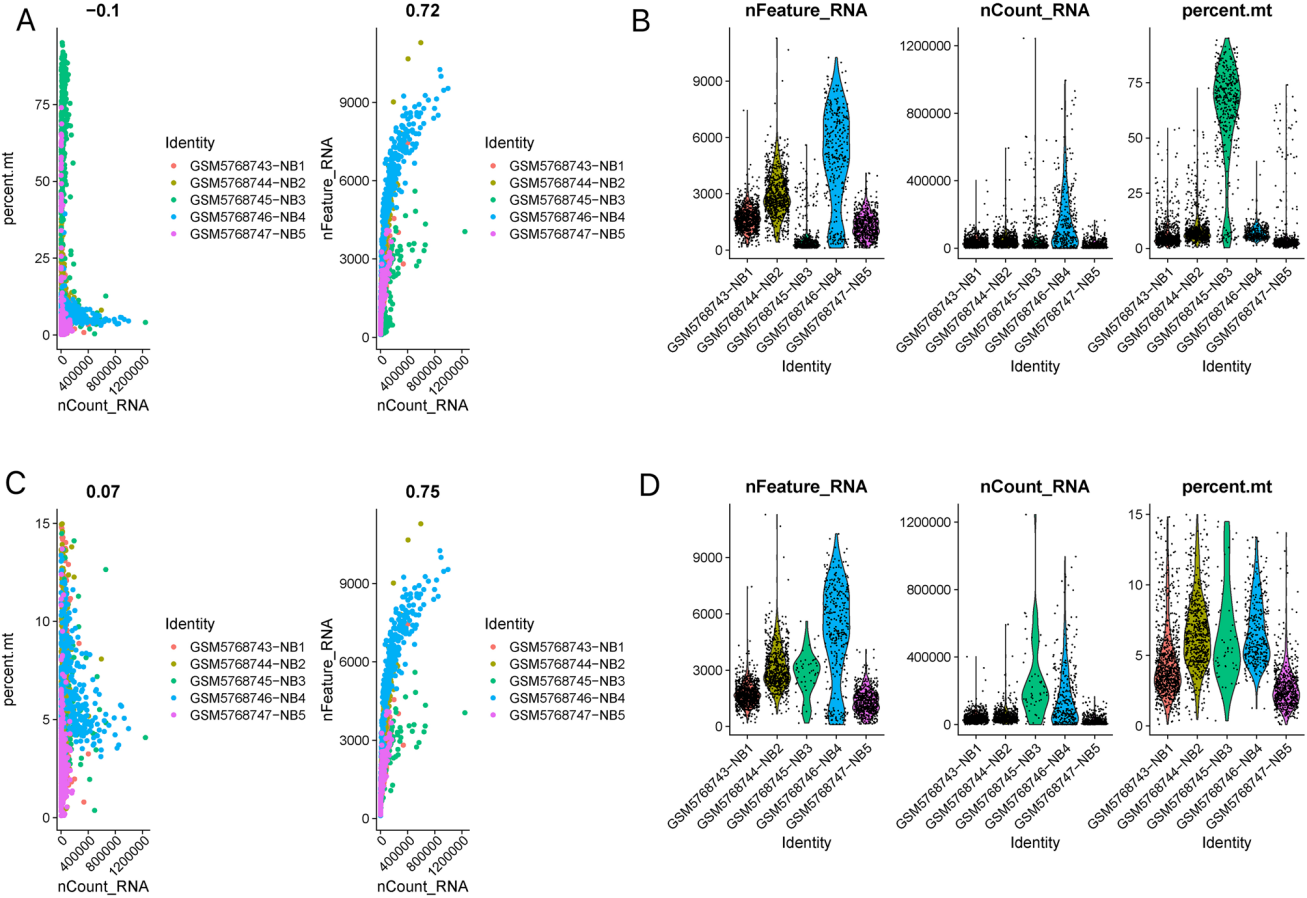
Pre-processing of single-cell expression profile data. (**A**, **B**) Five NB-related tissue samples, with 3169 cells examined for expression levels. (**C**, **D**) After quality control of scRNA-seq, 2594 core cells were identified.

A schematic representation of the study protocol has been shown in Fig. 1. My current analysis used expression profiles containing five NB-related tissue samples, with 3,169 cells examined for expression levels (Fig. 2A, B). Only cells with nFeature_RNA > 100 and percent.mt < 15 in the expression profile were retained for this analysis. Exactly 2,594 cells were included for subsequent analysis of expression levels of the feature (Fig. 2C, D).

**Figure 3:**
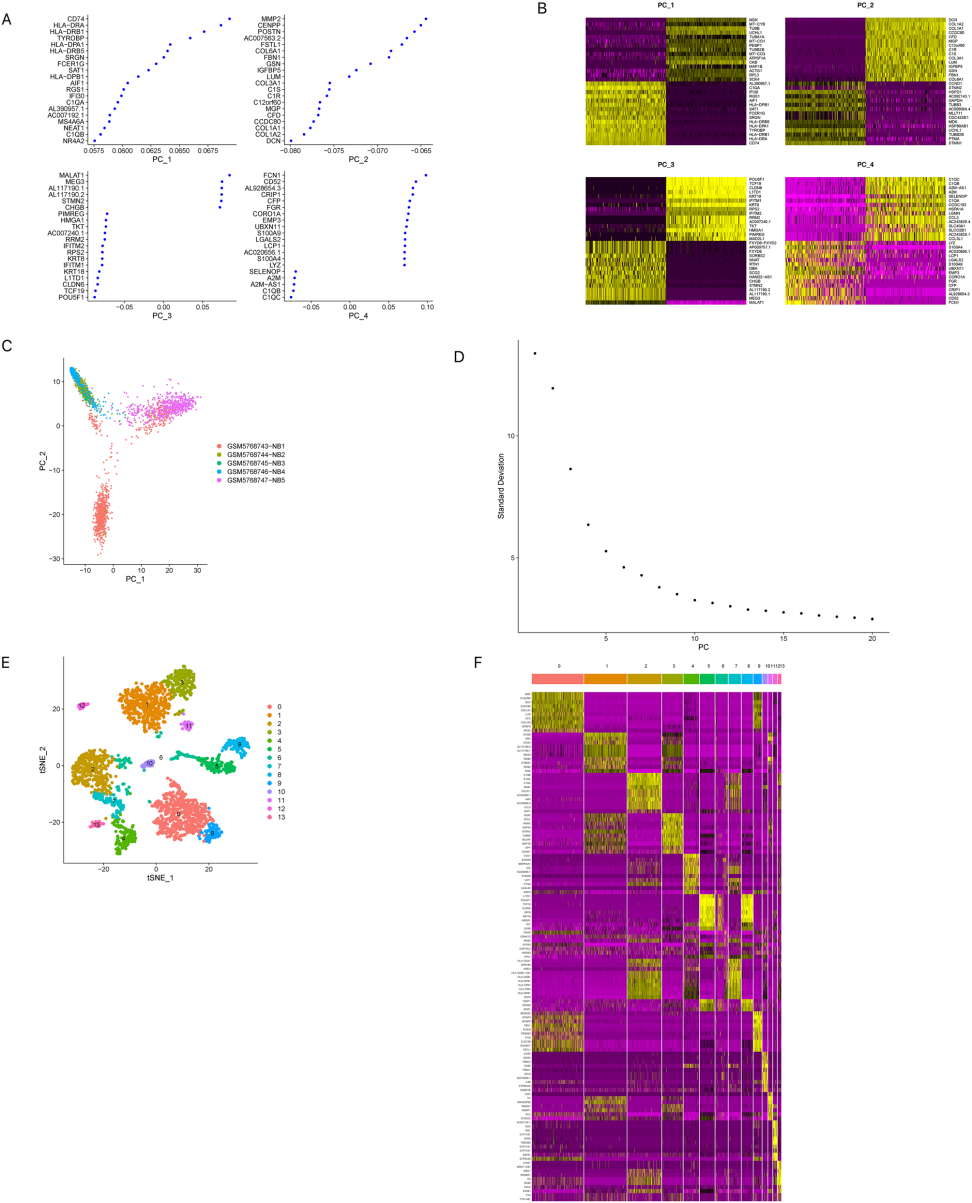
Subtype clustering analysis of single-cell samples. (**A**) PCA dimensionality reduction analysis on 20 genes. (**B**) PCA-heatmap. (**C**) The PCA was used to identify the significantly available dimensions of data sets. (**D**) The optimal number of PCs was 17. (**E**) Basing on the available significant components from PCA, I performed t-SNE algorithm and classified 14 cell clusters. (**F**) Heat map showing the expression levels of specific marker genes in each cluster.

The RunPCA function was used to reduce the dimensionality of cells with NCPs set to 20, which specifies how many principal components should be selected in the data set. The correlation between the genes characterized on each principal component and the respective principal component was plotted using the VizDimLoadings function, thereby indicating the relationship between 20 genes and the principal components. Through PCA dimensionality reduction analysis on 20 genes, I found that they had different scores in various dimensions (Fig. 3A, B). However, the PCA dimensionality reduction analysis between samples did not show significant overall differences (Fig. 3C). By observing the ElbowPlot, the optimal number of PCs was found to be 17 (Fig. 3D), The t-SNE algorithm was used to cluster cells and visualize the similarity between cells, similar cells were closer in the t-SNE plot, while dissimilar cells were further apart. Finally, 14 subtypes were obtained through t-SNE (Fig. 3E). I found many genes with widely varying expression levels between these subtypes and showed the expression levels of the 10 genes with the largest differences in expression levels between subtypes (Fig. 3F).

### Annotation of cluster subtypes and Analysis of receptor-ligand pairs

**Figure 4:**
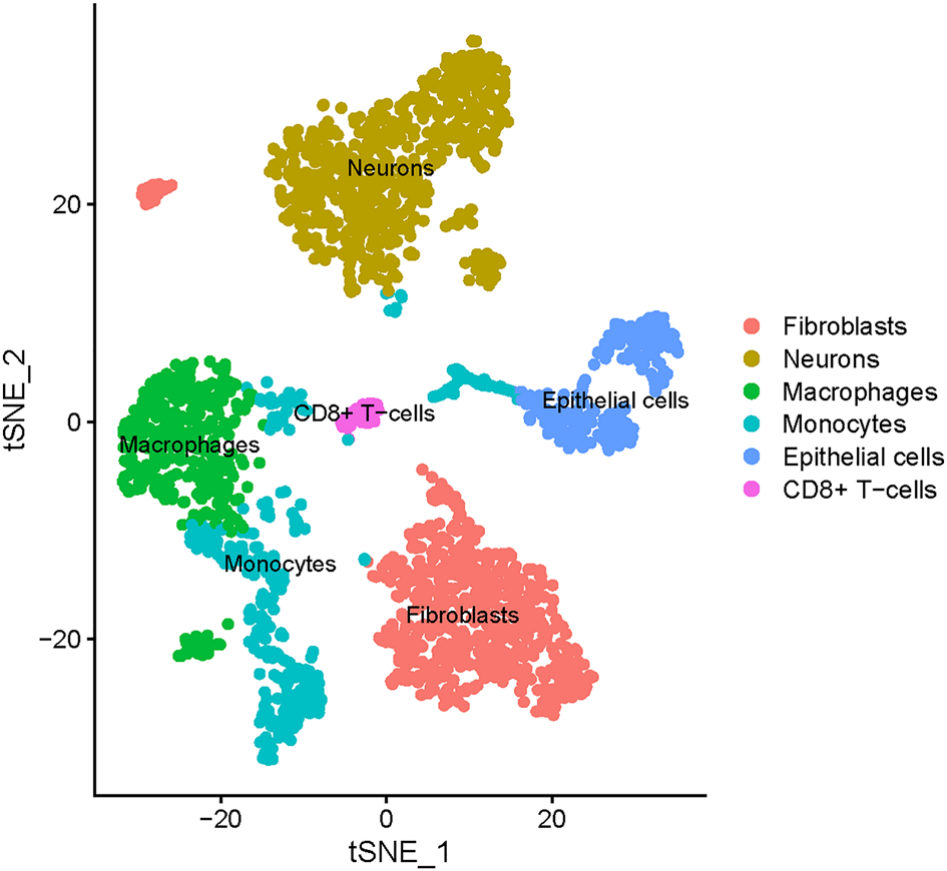
Fourteen clusters were assigned to six categories of cells: neurons, epithelial cells, fibroblasts, macrophages, monocytes, and CD8 + T-cells.

I used BlueprintEncodeData as the annotation data to annotate each subtype using the R package SingleR. Fourteen clusters were assigned to six categories of cells: neurones, epithelial cells, fibroblasts, macrophages, monocytes, and CD8 + T-cells (Fig. 4). NB is a neurological tumor that arises from neural crest (NC) cells. NC cells delaminate from the dorsal neural tube (NT) and migrate toward their destination^9^. Therefore, NB exhibits neuroepithelial properties. Neurons and epithelial cells maybe represent the tumor cell population; CD8 + T cells, macrophages, and monocytes represent the immune cell population; and fibroblasts represent the stromal cell population in NB. Finally, I extracted 3276 cell subtype marker genes from single-cell expression profiles using FindAllMarkers (Additional file 1).

**Figure 5:**
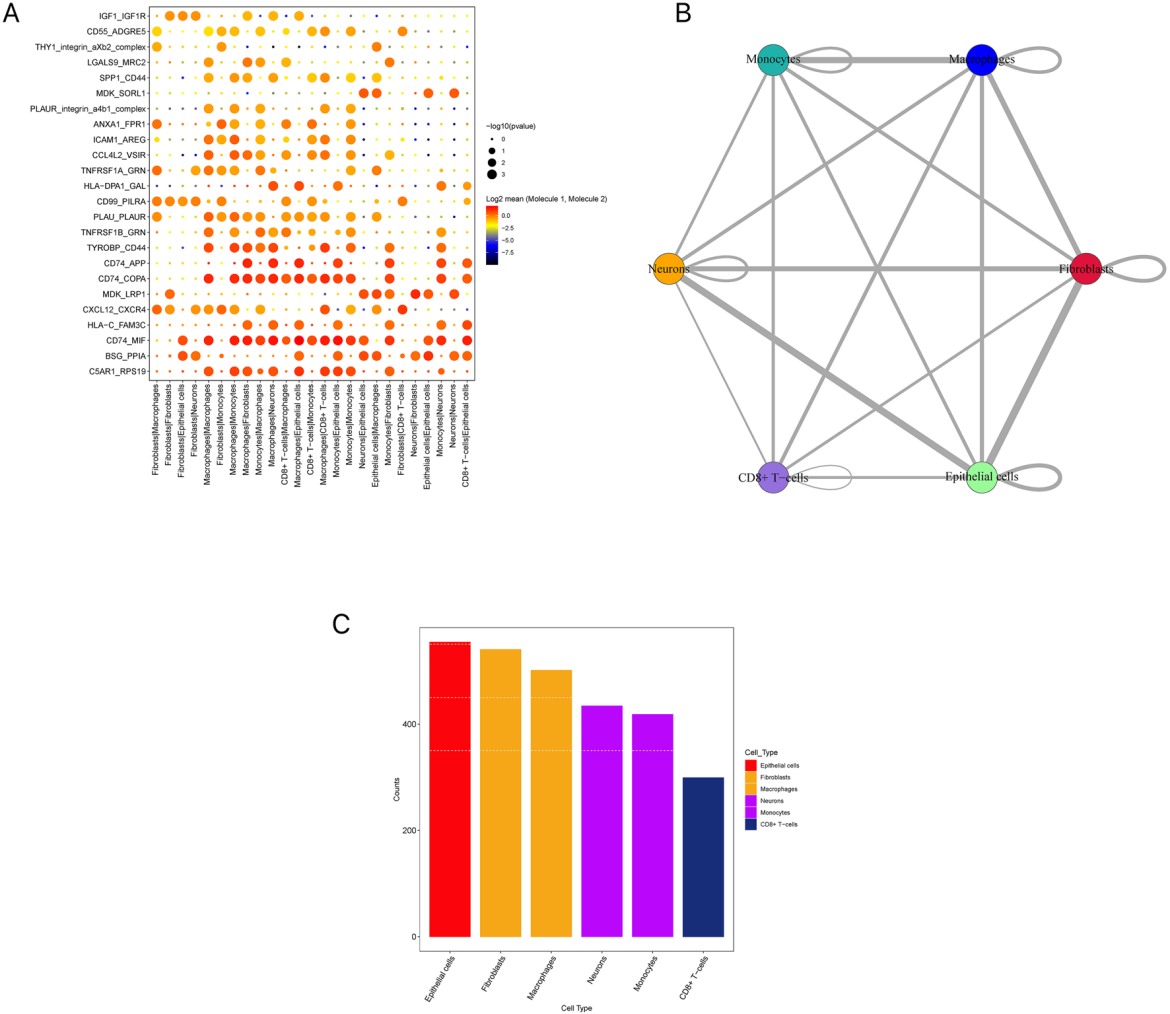
Analysis of receptor-ligand pairs. (**A**) Some ligand and receptor pairs for display. Macrophages, CD8 + T-cells, monocytes, and epithelial cells had high interaction scores with CD74_MIF and CD74_COPA. (**B**) The number of potential ligand-receptor pairs between macrophages, fibroblasts, epithelial cells, and other cells was found to be extremely high. (**C**) The subtype of epithelial cells had the highest number of potential interactions with other cell subtypes.

I used the software package CellphoneDB to analyse ligand-receptor relationships in single-cell expression profiles. Finally, I selected some ligand and receptor pairs for display. I found that macrophages, CD8 + T-cells, monocytes, and epithelial cells had high interaction scores with CD74_MIF and CD74_COPA (Fig. 5A). CD74(major histocompatibility complex [MHC] lass II invariant chain, II) is a non-polymorphic type II transmembrane glycoprotein. In addition to being a MHC class II chaperone, CD74 is a high-affinity cell membrane receptor for macrophage migration inhibitory factor (MIF) that regulates T-cell- and B-cell development, dendritic cell (DC) motility, macrophage inflammation, and thymic selection^10^. These results show that CD74 is closely related to epithelial cells, neurons, and immune cells in NB, suggesting that CD74 plays an important role in the occurrence and development of NB.

The number of potential ligand-receptor pairs between macrophages, fibroblasts, epithelial cells, and other cells was also extremely high (Fig. 5B). Finally, I counted the number of ligand-receptor gene pairs corresponding to each cell group. I found that the subtype of epithelial cells had the highest number of potential interactions with other cell subtypes (Fig. 5C).

### Functional analysis of marker genes for key subtypes

**Figure 6:**
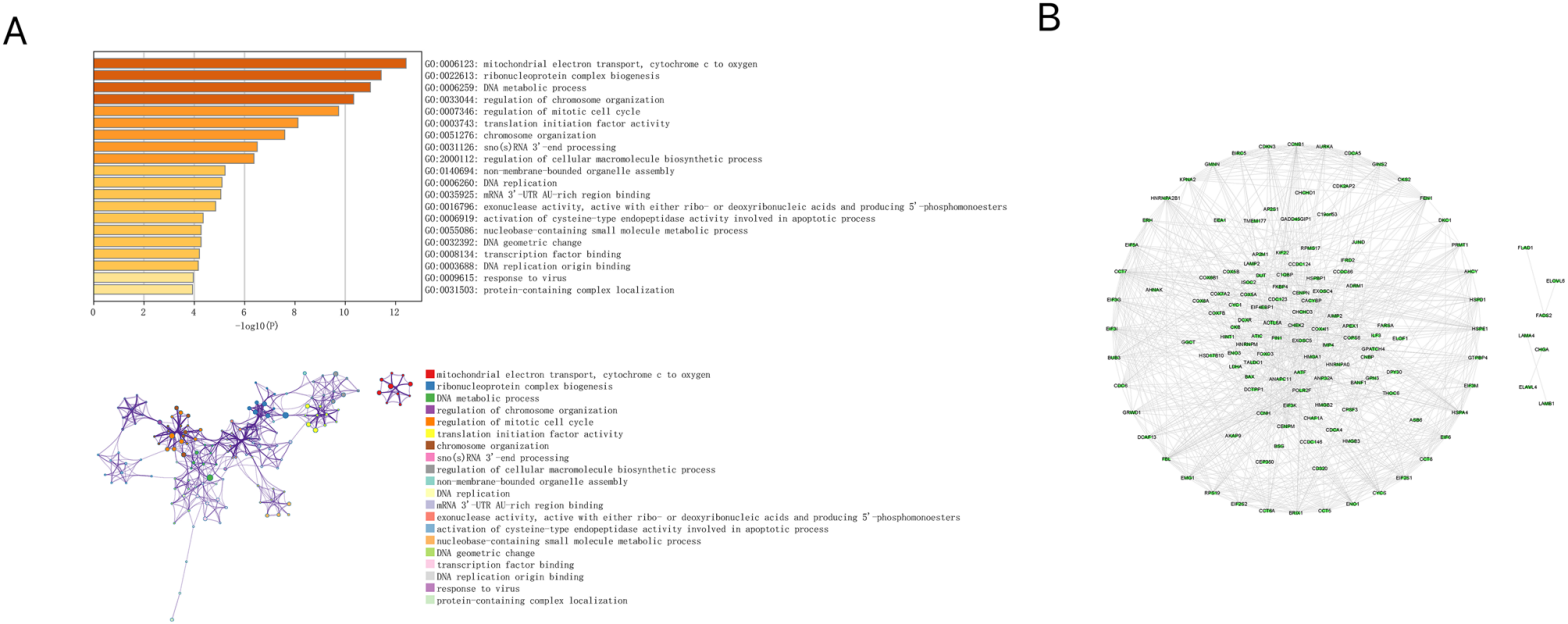
Functional analysis of marker genes for key subtypes. (**A**) Pathway analysis of prognostic genes with the Metascape database. (**B**) Protein interaction network analysis of genes in the prognostic gene set by the Cytoscape software.

To further identify key genes in the subtype marker gene set for epithelial cells, I collected clinical information on patients with NB and screened 154 prognosis-related genes using Cox univariate regression. Further analysis of the prognostic genes pathways with the Metascape database showed that these marker genes were primarily enriched for mitochondrial electron transport, ribonucleoprotein complex biogenesis, DNA metabolic processes, regulation of chromosome organization, DNA replication, and other pathways (Fig. 6A). These pathways are involved in the cell cycle and proliferation process. Furthermore, mitochondrial electron transport is required for tumor initiation, growth, and metastasis^11^.

I also performed a protein interaction network analysis of genes in the prognostic gene set by Cytoscape software (Fig. 6B). Most genes in the network diagram such as *BIRC5* (survivin), cyclin-dependent kinase inhibitor 3 (*CDKN3*), *CCNB1*, *AURKA*, and eukaryotic translation initiation factor 4E-binding protein 1 (*EIF4EBP1*) play important roles in the development and metastasis of NB^12–14^.

### Obtaining prognosis-related genes and constructing a predictive model

**Figure 7:**
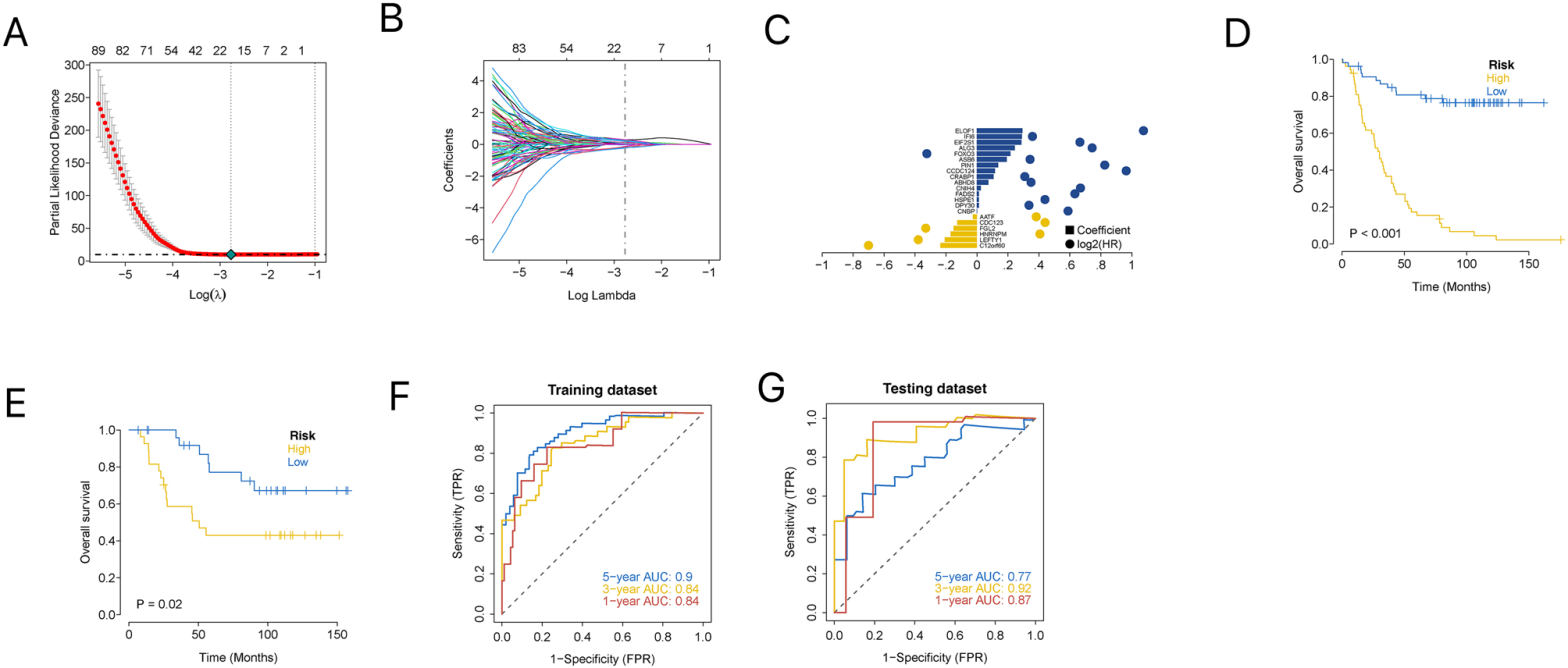
Lasso analysis and model construction. (**A**) A coefficient profile plot was generated against the log (lambda) sequence. Selection of the optimal parameter (lambda) in the LASSO model for TARGET. (**B**) LASSO coefficient profiles of the 21 IRGs in TARGET-NB. (**C**) Lasso Coefficient HR. (**D**) Kaplan–Meier survival curve analysis in the high-risk and low-risk NB patients in the training subset. (**E**) Kaplan–Meier survival curve analysis in the high-risk and low-risk NB patients in the testing subset. (**F**) Time-dependent ROC curve for 1-year, 3-years, and 5-years prediction (training subset). (**G**) Time-dependent ROC curve for 1-year, 3-years, and 5-years prediction (testing subset).

To further identify key genes in the prognostic gene set, we used the feature selection algorithm of Lasso regression to screen for characteristic genes in the NB (Fig. 7A–C). I randomly divided the TARGET patients into training and internal validation sets in a 2:1 ratio. After the Lasso regression analysis, I obtained the best risk score value for each sample for subsequent analyses (risk score = C12orf60 × (−0.236733770190884) + LEFTY1 × (−0.208282329754236) + HNRNPM × (−0.169755802627903) + FGL2 × (−0.151331192024352) + CDC123 × (−0.127307193600985) + AATF × (−0.0267384340199854) + CNBP × 0.00146836752628189 + DPY30 × 0.0114982992406702 + HSPE1 × 0.0115376558208352 + FADS2 × 0.012440259330444 + CNIH4 × 0.0240070849825049 + ABHD8 × 0.0742322217111553 + CRABP1 × 0.106066696134723 + CCDC124 × 0.116166397537017 + PIN1 × 0.136354334440786 + ASB6 × 0.192414976174186 + FOXO3 × (0.214884239283954 + ALG3 × 0.24364248402027 + EIF2S1 × 0.287257489058717 + IFI6 × 0.290358384842998 + ELOF1 × 0.292519860625507). The patients were divided into high- and low-risk groups based on the median risk score and analysed using Kaplan − Meier curves. The overall survival (OS) of the high-risk group was significantly lower than that of the low-risk group in both the training and test sets (Fig. 7D–E). Furthermore, the results of the ROC curves in both the training and test sets indicated that the model had good validation performance (Fig. 7F, G).

**Figure 8:**
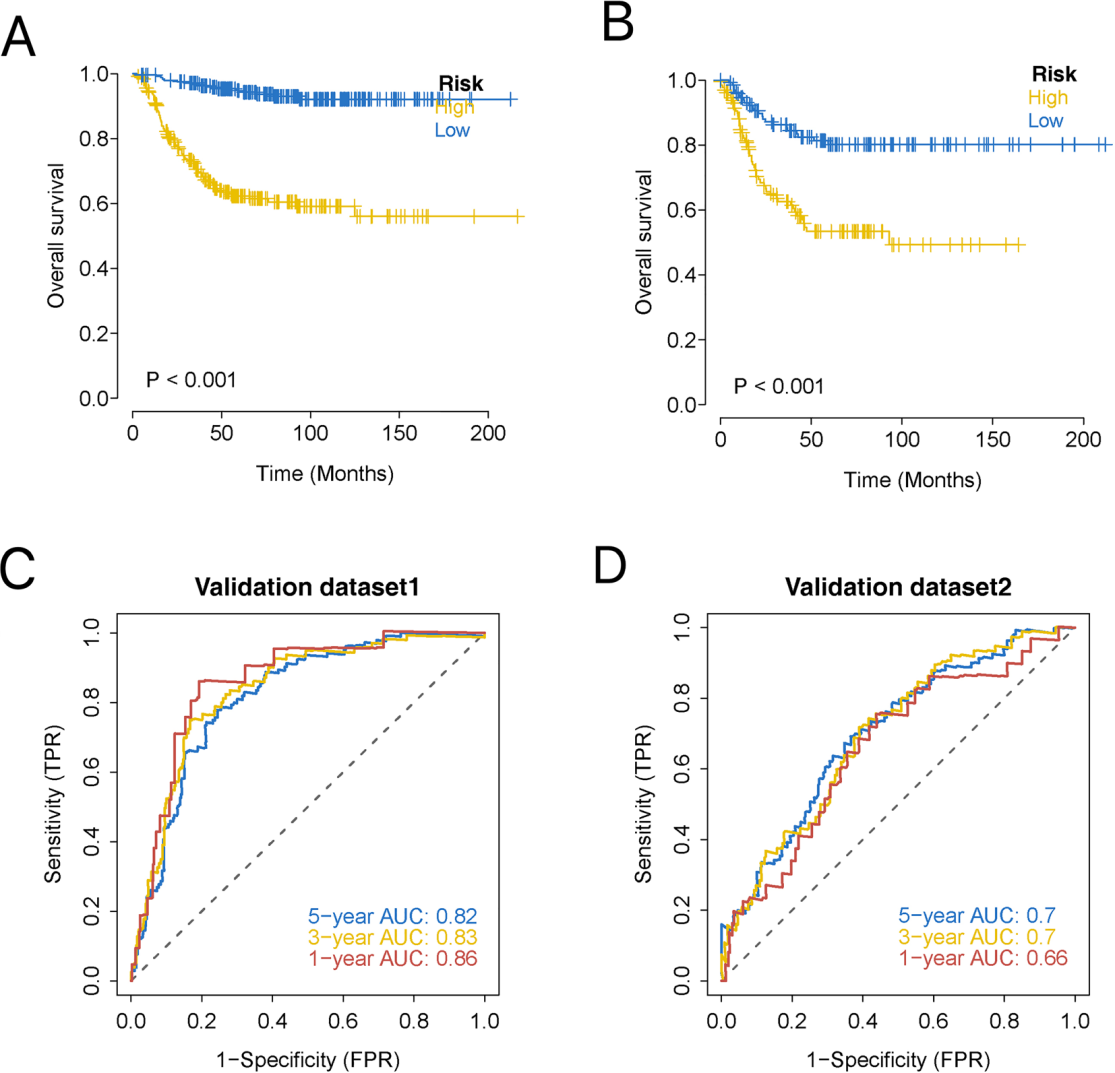
Validation of the robustness of the prognostic model using external datasets. (**A**) KM for validation set1; (**B**) KM for validation set2; (**C**) Survival ROC for external validation dataset1; (**D**) Survival ROC for external validation dataset2.

I downloaded processed data with survival information from public databases for patients with NB (GSE62564 and GSE85047) and predicted the clinical classification of the patients in the GEO database using the model. I evaluated the survival differences between the two groups using Kaplan − Meier analysis to investigate the stability of the predictive model. The results showed that the OS of the two GEO external validation sets was significantly lower in the high-risk group than in the low-risk group (Fig. 8A, B). To validate the accuracy of the model, I performed a ROC curve analysis on the model using an external dataset. The results showed that the model had strong predictive power to predict the prognosis of patients (Fig. 8C, D).

### Multi-omics study to explore the clinical predictive value of the model

**Figure 9:**
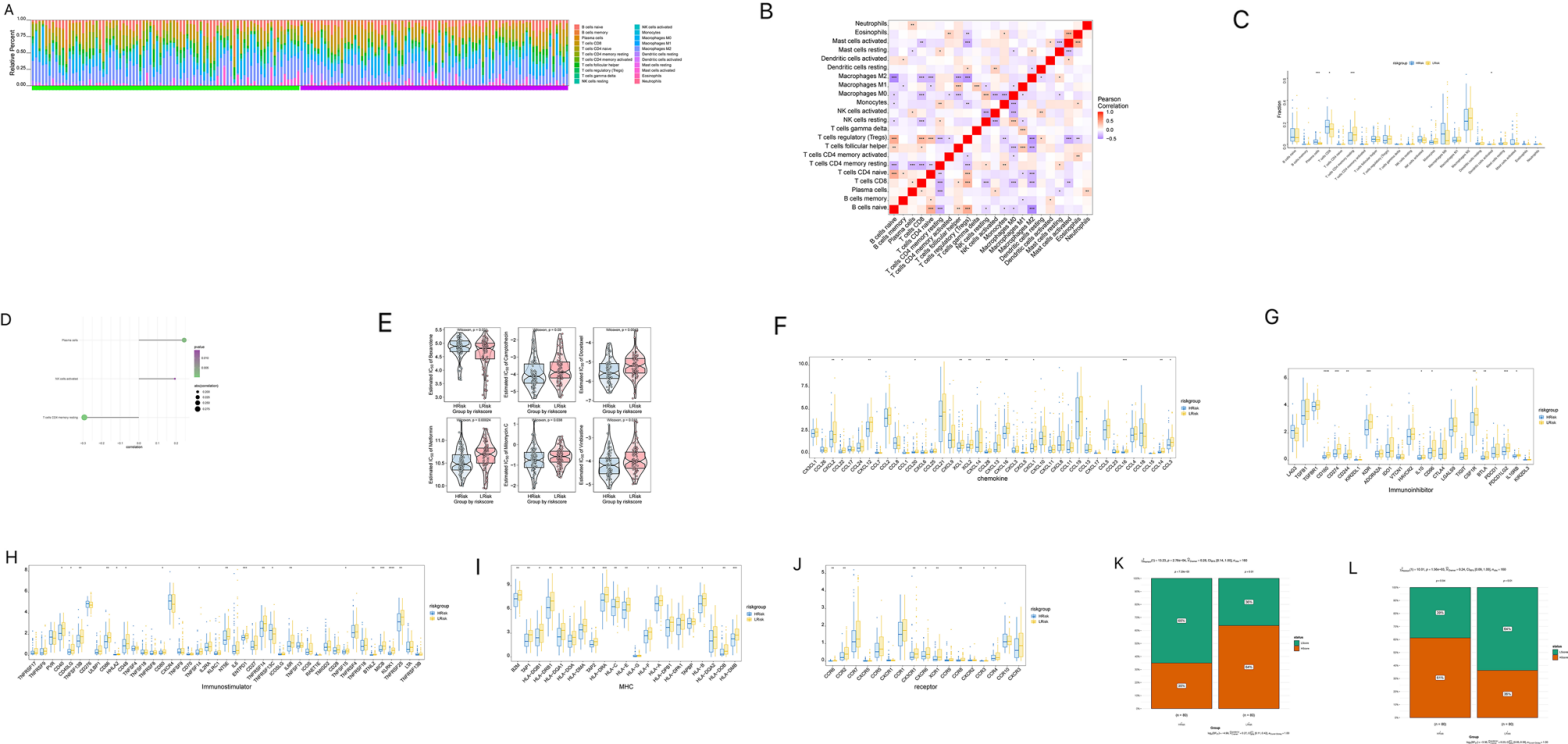
Multi-omics analysis of the clinical predictive value of the model. (**A**) Stacked bar chart of the distribution of 22 immune cells in each NB sample of the TARGET cohort. (**B**) Pearson correlation between immune cells, red for positive correlation, purple for negative correlation. (**C**) Differences in immune cell counts between the high-risk and low-risk groups. (**D**) The correlation between the risk score and the immune cells, the circle size indicates the strength of the correlation, and the color indicates the *p*-value. (**E**) The difference on the therapeutic sensitivities of six chemotherapy drugs. (**F**) Chemokine association. (**G**) Immuno-inhibitor association. (**H**) Immuno-stimulator association. (**I**) MHC association. (**J**) Receptor association. In the analysis of Dysfunction (**K**) and Exclusion (**L**), the H-score group refers to samples with scores higher than the median score; the L-score group refers to samples with scores not higher than the median score.

The tumour microenvironment (TME) consists primarily of tumour-associated fibroblasts, immune cells, extracellular matrix, various growth factors, inflammatory factors, specific physicochemical characteristics, and cancer cells. The TME significantly influences the diagnosis, survival outcome, and chemotherapy sensitivity of tumours. The relationship between risk score and tumour immune infiltration was analysed to further explore the potential molecular mechanisms by which risk scores affect NB progression. My results showed that the distribution of the immune levels of the different immune factors in the samples was not entirely consistent (Fig. 9A). There were multiple significantly correlated pairs of immune factors (Fig. 9B). The aggregation of CD4 + T cells and CD8 + T cells enhanced immune capacity and anti-tumor activity in NB. NK cells are a type of lymphocyte that possess cytotoxic activity and can effectively respond to the presence of a variety of tumor cells ^15^. Compared to the high-risk group, the levels of immune factors, such as resting memory CD4 T cells, were significantly higher in the low-risk group, while the levels of immune factors, such as plasma cells and CD8 T cells, were significantly lower (Fig. 9C). The risk scores were positively correlated with plasma and activated NK cells and negatively correlated with resting memory CD4 T cells (Fig. 9D).

The effects of early-stage NB surgery combined with chemotherapy are well established. I used the R package ‘pRRophetic’ to predict the chemo-sensitivity of each tumour sample based on drug sensitivity data from the GDSC database to further explore the risk scores and sensitivity of commonly used chemotherapy drugs. My results showed that high-risk scores were significantly correlated with the sensitivity of patients to drugs such as bexarotene, camptothecin, docetaxel, metformin, mitomycin C, and viNBastine (Fig. 9E).

I extracted multiple sets of immune-related genes from the tumour-immune system interaction database (TISIDB), including immunomodulators, chemokines, and cellular receptors. I found that the expression levels of many immune-related genes were significantly different between the high-risk and low-risk groups. The expression levels of chemokines, immune modulators, MHC molecules, and cell receptor molecules were lower in the high-risk group than in the low-risk group (Fig. 9F–J).

Furthermore, the analysis of tumour immune dysfunction and exclusion revealed differences between the high-risk and low-risk groups, with dysfunction and exclusion significantly different. Dysfunction scores are generally used to assess the degree of cellular or organ functional abnormality, which is often associated with disease progression and prognosis^16^. Generally speaking, the higher the Dysfunction score, the poorer is the prognosis. Interestingly, however, we found that the high-risk group had a lower Dysfunction score than the low-risk group (Fig. 9K). We suspect that this unexpected finding could be attributed to the fact that prognostic assessments are often influenced by a combination of factors. The Dysfunction score is just one of a number of potential indicators of prognosis, and other factors, including disease stage, molecular subtype, and treatment regimen, may also have a significant influence on prognosis. Consequently, although Dysfunction scores were lower in the high-risk group, other factors may have played a more important role in the prognostic model.

The Exclusion score is generally associated with the role of T cells (or other immune cells) in the immune system in mediating anti-tumor or anti-pathogen immunity. A high Exclusion score indicates that immune cells are excluded from the vicinity of a tumor or pathogen, or have difficulty in entering a lesioned area. This could indicate that the capacity of the immune system to attack tumor cells or pathogens is limited or suppressed, thereby resulting in a potential attenuation of the immunological inhibition of tumor growth and proliferation, thus contributing to a poorer prognosis ^17^. For certain types of tumor, high Exclusion scores are associated with immune escape and evasion of immune surveillance. Tumor cells may evade immune system attack by inhibiting the entry of T cells or by preventing T cells from functioning within the tumor. This may in turn promote tumor growth and progression, thereby increasing the difficulty of treatment. Consequently, high Exclusion scores are typically correlated a poorer prognosis^17^. Our model revealed that the high-risk group had higher Exclusion scores than the low-risk group (Fig. 9L).

Collectively, the findings of the afore-mentioned immunoassays reveal that the high-risk group in our model has a limited effect on immunotherapy.

### Correlation analysis between the risk of onset with independent prognosis and multiple clinical indicators

**Figure 10:**
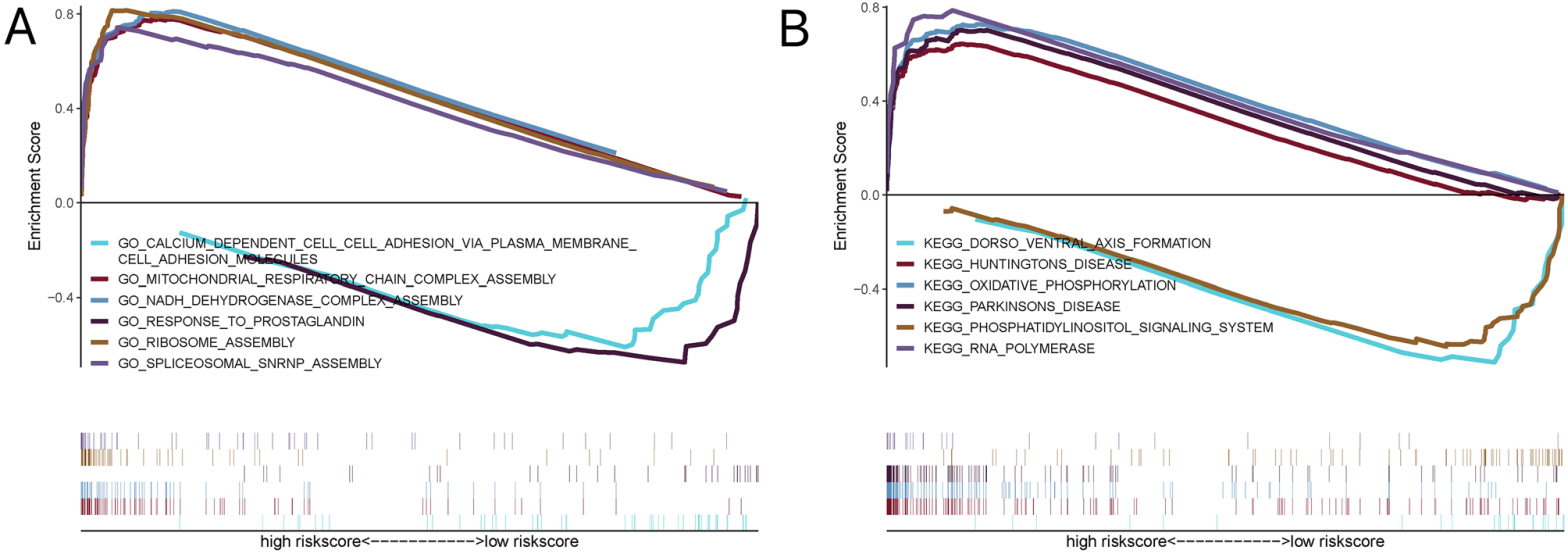
GSEA analysis of risk scores. (**A**) GO pathways showed that “ribosome assembly,” “NADH dehydrogenase complex assembly,” “mitochondrial respiratory chain complex assembly,” and “spliceosomal snRNP assembly” were significantly enriched in the high-risk group. (**B**) KEGG pathways showed that “RNA polymerase” and “oxidative phosphorylation” were significantly enriched in the high-risk group.

I examined the specific signalling pathways involved in high-risk and low-risk related models and explored the potential molecular mechanisms by which risk scores influence tumour progression. GO analysis showed that “ribosome assembly,” “NADH dehydrogenase complex assembly,” “mitochondrial respiratory chain complex assembly,” and “spliceosomal snRNP assembly” were significantly enriched in the high-risk group, whereas “calcium dependent cell adhesion via plasma membrane cell adhesion molecules” and “response to prostaglandin” were significantly enriched in the low-risk group (Fig. 10A). According the KEGG pathway, “RNA polymerase” and “oxidative phosphorylation” were significantly enriched in the high-risk group, whereas “dorso-ventral axis formation” and “phosphatidylinositol signaling system” were significantly enriched in the low-risk group (Fig. 10B). I presented some of the highly significant pathways in a focused manner, suggesting that perturbations of these signalling pathways in patients in the high-risk and low-risk groups affect the prognosis of patients with NB.

**Figure 11:**
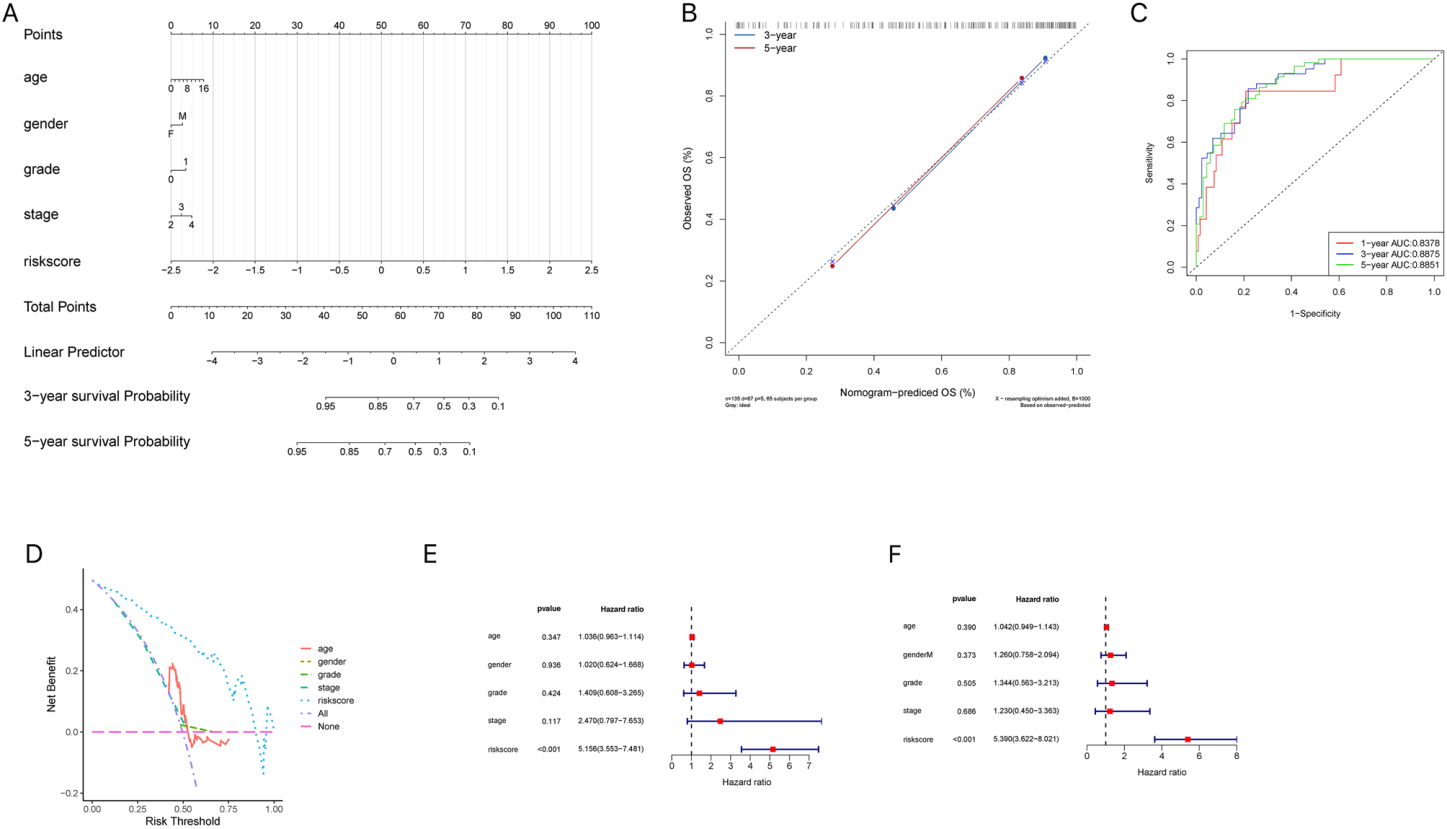
Risk of onset and independent prognosis analysis. (**A**) The nomogram for predicting the 3- and 5- years OS of NB patients. (**B**) The calibration curve of the nomogram for predicting 3- and 5-years OS of NB patients. (**C**) Time-dependent ROC curve for 1-year, 3-years, and 5-years prediction. (**D**) Decision curve analysis (DCA). (**E**) Univariate Cox regression analysis. (**F**) Multifactorial Cox regression analysis.

I integrated the clinical information as well as the risk scores of patients in the high-risk and low-risk groups and displayed the results of the regression analysis in the form of a nomogram, where the results of the logistic regression analysis showed that in all my samples, the distribution of the values of the different clinical indicators of NB and the risk score values contributed to several scoring processes (0, undifferentiated or poorly differentiated; 1, differentiating in the grade indicator). Of these, only the distribution of the risk score values derived from the model analysis contributed throughout the scoring process of the predictive analysis (Fig. 11A). We also performed a predictive analysis of OS for the 3-year and 5-year periods of NB, the results of the calibration curve and ROC curve of the nomogram showed a reliable performance, with an AUC of 0.8378, 0.8875, and 0.8851 at 1, 3, and 5 years, respectively (Fig. 11B, C). As shown in Fig. 11D, compared with the age, gender, grade and stage indexes, the new riskscore provided greater net benefits both in the derivation and validation cohorts. Simultaneously, through univariate and multivariate analyses, I found that the risk score was an independent prognostic factor for patients with NB (Fig. 11E, F).

**Figure 12:**
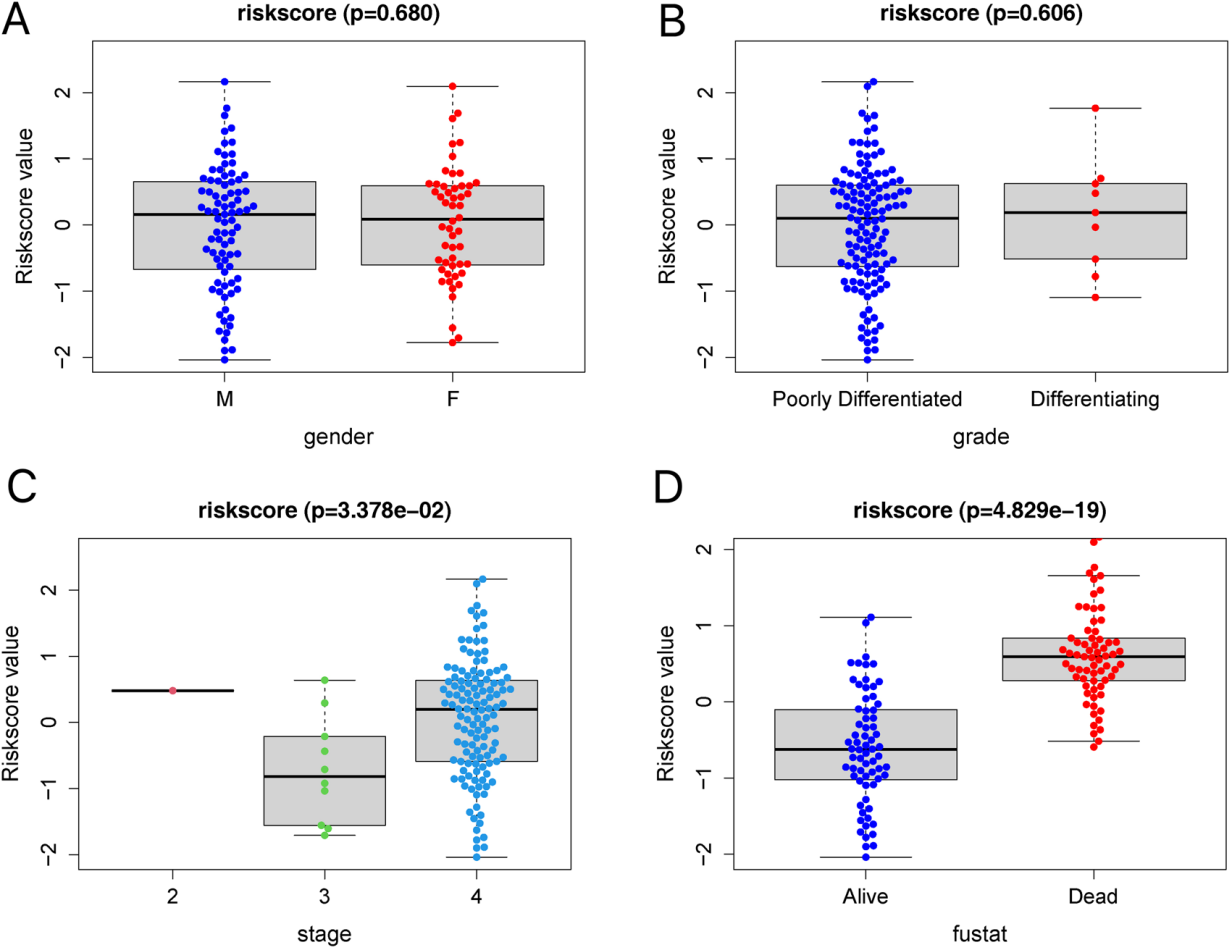
Correlation analysis of risk scores with clinical characteristics. (**A**) Relationship between gender with risk scores. (**B**) Relationship between grade with risk scores. (**C**) Relationship between stage with risk scores. (**D**) Relationship between fustat with risk scores.

I grouped the risk score values of all samples by different clinical indicators, including gender, grade, stage, and fustat, and presented them as box plots (Fig. 12A–D). Through the rank-sum test, I found that these risk scores were significantly different between the groups for the two clinical indicators, stage and fustat (*P* < 0.05). High-risk scores were positively correlated with high tumor staging and patient mortality in NB.

### Study of gene expression levels in diseases and Analysis of regulatory networks of model genes

**Figure 13:**
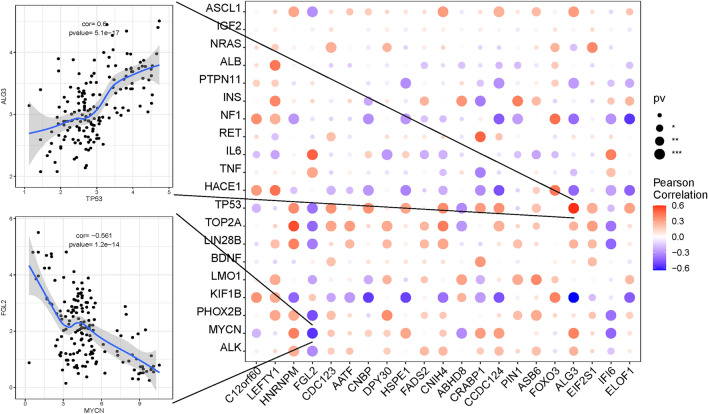
The expression levels of the model genes were significantly correlated with the expression levels of several NB-related genes.

I used the GeneCards database (https://www.genecards.org/) to obtain NB-related disease genes in 5294 children and analysed the expression levels of the 21 model genes and the top 20 genes in the relevance score. I found that the expression levels of the model genes were significantly correlated with the expression levels of several disease-related genes. For example, in a prognostic model, *ALG3* expression was positively correlated with *TP53* and *MYCN*, whereas *FGL2* expression was negatively correlated with *ALK*, *MYCN*, *PHOX2B*, and *LIN28B*. *HNRNPM* expression was positively correlated with *ALK*, *MYCN*, *PHOX2B*, *LIN28B*, *TOP2A*, and *TP53* (Fig. 13).

**Figure 14:**
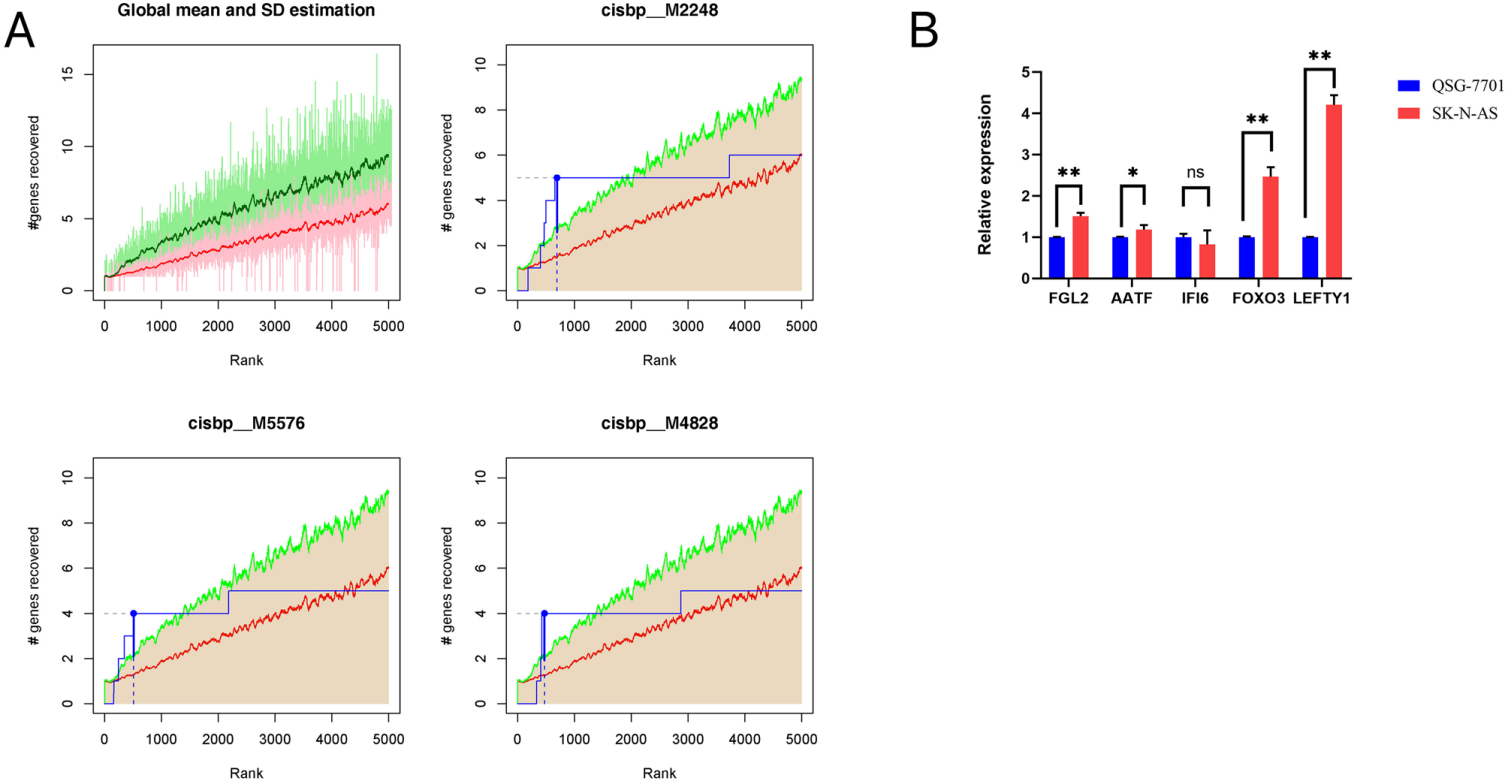
Analysis of regulatory networks of model genes. (**A**) Enrichment analysis was performed on these transcription factors using cumulative recovery curves. Motif-TF annotation and selection of important genes were carried out. (**B**) The expression of AATF, FGL2, FOXO3, IFI6 and LEFTY1 in SK-N-AS and QSG-7701 by qPCR.

**Figure 15:**
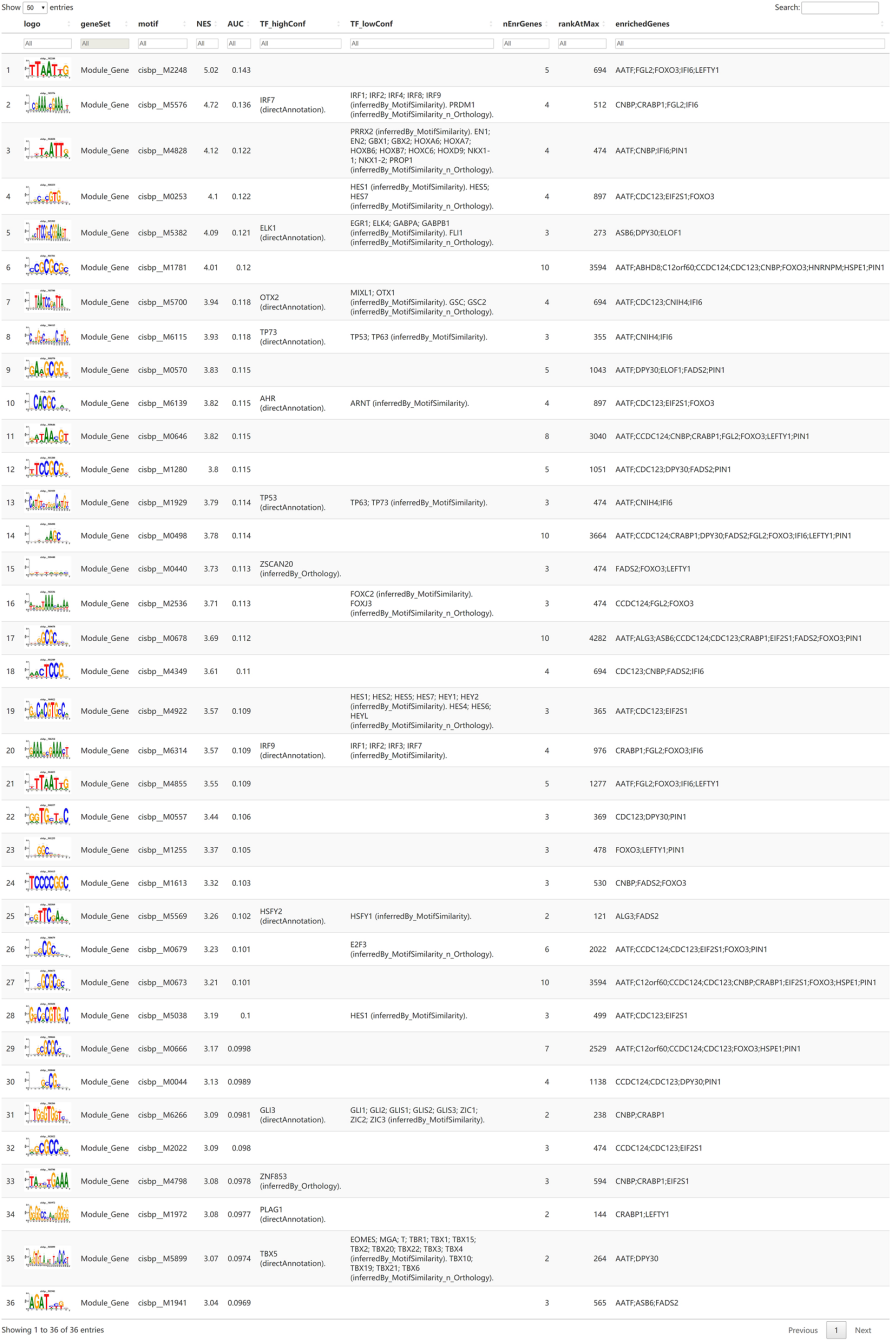
All the enriched motifs and corresponding transcription factors of the model genes.

I used 21 model genes as the gene set for this analysis and found that they were regulated by multiple transcription factors and other common mechanisms. Therefore, an enrichment analysis was performed on these transcription factors using cumulative recovery curves (Fig. 14A). Motif-TF annotation and selection of important genes were carried out. The analysis showed that the motif annotation with the highest NES: 5.02) was cisbp__M2248. Five genes were enriched in this motif, i.e., *AATF, FGL2, FOXO3, IFI6,* and *LEFTY1*. I further assessed the expression of *AATF, FGL2*, *FOXO3, IFI6,* and *LEFTY1* in the NB cell line (SK-N-AS) and the healthy liver cell line (QSG-7701) by qPCR. As shown in Fig. 14B, four were differentially expressed in NB cells among the five model genes and normal live cells, i.e., *AATF, FGL2*, *FOXO3*, and *LEFTY1*. The expression levels of these four genes were upregulated in NB and the expression level of *IFI6* was very low. I displayed all enriched motifs and corresponding transcription factors from the model genes (Fig. 15).

## Discussion

The TME is a complex network system comprised of seven parts: the hypoxia niche, immune microenvironment, metabolic microenvironment, acidic niche, innervated niche, mechanical microenvironment, and microbial microenvironment^18^. Intercellular communication is divided into direct and indirect communication. Direct contact communication involves gap junctions, tunneling nanotubes, and LRIs, whereas indirect communication involves exosomes, apoptotic vesicles, and soluble factors^19^. Intercellular communication mediates the crosstalk between the TME and the host, as well as between cells and cell-free components, causing changes in the tumor hallmarks of the TME. This includes changes in tumor cell proliferation, invasion, apoptosis, angiogenesis, metastasis, inflammatory response, gene mutation, immune escape, metabolic reprogramming, and therapeutic resistance^19^. LRIs are the most important form of intercellular communication. It is reported that most cells express from tens to hundreds of ligands and receptors, forming a highly connected signal network through multiple ligand receptor pairs^20^. The biological importance and druggable properties of receptors and their corresponding ligands have designated them as especially useful clinical targets for cancer^21^. Checkpoint inhibitors that operate based on the ligand—receptor interaction have become powerful tools for clinical therapy^21^. Therefore, there are broad prospects for the research of intercellular communication inference and receptor-ligand pairs in the field of molecular oncology.

To improve therapeutic strategies for malignant tumors, the communication between various cell types in the TME must be quantified. Intercellular communication inference methods primarily include network-based, machine learning-based, and spatial information-based approaches^22^. Network-based methods explore network algorithms for decoding intercellular communication. Hou et al.^23^ developed the Network Analysis Toolkit for Multicellular Interactions to score intercellular communication. Machine-learning-based methods exploit machine-learning models and algorithms to measure the communication specificity between two cell types. Cillo et al.^24^ developed a deterministic annealing Gaussian mixture model-based clustering algorithm to assess the communication between immune cells and carcinogen- and virus-induced cancers. Spatial information-based methods use scRNA-seq data, LRIs, and spatial information to infer intercellular communication. Li et al.^25^ presented a bivariant Moran’s statistical model (SpatialDM) to detect spatially co-expressed ligand and receptor pairs, their local interacting spots (single-spot resolution), and communication patterns. Recently, a Boosting-based LRI prediction method (CellEnBoost) was developed for intercellular communication elucidation based on an Ensemble of Light gradient boosting machine (Light GBM) and AdaBoost, combined with a Convolutional Neural Network (AdaBoost-CNN)^26^. Peng et al.^27^ developed an ensemble deep-learning framework, CellComNet, to decipher ligand receptor-mediated intercellular communication using single-cell transcriptomic data. CellComNet was compared with other four competing protein–protein interaction prediction models to obtain the best area under the curve (AUC) and area under the precision-recall curve (AUPR) on the four LRI datasets, elucidating the optimal LRIs classification ability. In this study, after evaluating a few software packages and considering their benchmark performances, the CellphoneDB software package was used to analyze the LRIs in single-cell expression profiles.

In this study, I used scRNA-seq data to analyze cell–cell LRIs in the TME of NB to identify key ICAGs genes. scRNA-seq data from 3169 cells of five NB samples were analysed to explore the intratumor heterogeneity of NB by exploring cell clusters. Analysis of the scRNA-seq data from the 2594 cells obtained revealed that 14 cell clusters belonged to six cell types, involving broad cell types such as CD8 + T-cells, epithelial cells, fibroblasts, macrophages, monocytes, and neurones.

In recent years, the spatial transcriptome technique has been one of the major breakthroughs in the field of bioinformatics. This technique makes up for the defect that single-cell sequencing technology is difficult to measure the positional relation between individual cells by simultaneously measuring the spatial position and intracellular transcriptome data of a great number of cells, thus providing a new data basis for understanding the interactions between multiple cells. In 2022, Li et al.^28^ proposed cell clustering for spatial transcriptomics (CCST) based on graph neural networks (GNNs). CCST can determine the cell cycle stage of different cells within the same cell population and identify cell subtypes with new functions. The authors found that mice neuroblasts contain two clusters of cells: C0 and C1. Compared with C0, the C1 neuroblasts are spatially closer to mitral valve/cluster cells, endothelial cells and olfactory ensheathing cells (OECs). The analysis of the GO pathway showed that the C0 cells are associated with neural functions, such as the modulation across synaptic signaling pathways, suggesting that C0 calls represent functionally mature nerve cells, while C1 cells are immature cells. GO analysis showed that C1 cells are associated with cell adhesion. Their study had some implications for the occurrence of NB. It was also found in my study that some cells are likely to be the two groups of cell types of NB tumor cells: neurons and epithelial cells.

When analyzing the receptor–ligand relationship pairs, it was revealed that CD74 was closely related to neurons and epithelial cells. CD74 (MHC class II invariant chain, II) is a non-polymorphic type II transmembrane glycoprotein. Cell surface proteins play crucial roles in regulating cell-to-cell communication and interactions with the extracellular environment^29^. Hu et al.^29^ recently introduced a novel analytical framework called the gene function and protein association (GFPA) to explore the relationship between cell surface proteins and gene function. GFPA can also be used as an analytical tool to further explore whether the membrane protein CD74 can be used as a new potential therapeutic target and biomarker for predicting NB prognosis.

Interaction networks were created for the 12 cell types using CellphoneDB, the most widely used tool for studying intercellular interactions. I then performed a statistical analysis of the number of ligand-receptor gene pairs corresponding to each cell group and found that the epithelial cell subtype had the highest potential interactions with other subtypes. To further identify key genes in the epithelial cell subtype marker gene set, I collected clinical information from patients with NB and screened 154 prognosis-related genes using Cox univariate regression. I also performed a protein–protein interaction network analysis of genes in the prognostic gene set using Cytoscape software. Subsequently, I used the feature selection algorithm with Lasso regression to find characteristic genes in NB and developed a 21-gene prognostic model. Then, I analysed the relationship between the model and clinical characteristics, validated its clinical predictive value, and analysed the overall survival (OS) of each group based on the score calculated by the 21-gene prognostic model. Survival analysis using the Kaplan − Meier method showed that the OS of the high-risk group in both training and test sets was significantly lower than that of the low-risk group. Furthermore, the ROC curve analysis showed a strong predictive power of the model for the prognosis of the patient. Univariate and multivariate analyses revealed that the model’s risk score was an independent prognostic factor for patients with NB, and the rank-sum test found significant differences in the model’s risk score between clinical indicators, stage, and clinical status. Sun et al.^30^ established a new deep learning algorithm based on a graph convolutional network (GCN) with a graph attention network (GAT) (GCNAT) to predict metabolite–disease associations. A fivefold cross-validation shows outstanding AUC (0.950) and AUPR (0.405) of GCNAT when compared to previous methods and similar approaches. Compared to existing state-of-the-art methods, the proposed method achieves a higher predictive accuracy. In future studies, GCNAT will be used to test the predictive performance of tumor-related prognostic models.

By analysing the relationship between risk scores and tumour immune infiltration, I found that the distribution of immune levels of different immune factors in the samples was inconsistent. Compared to significantly higher levels of immune factors, such as resting CD4 T cells in the low-risk group samples, the high-risk group was associated with high levels of plasma cells, CD8 T cells, and activated NK cells, indicating an activated immune state. Increasing evidence suggests that activation of immune cells (e.g., CD4, CD8, and NK cells) can kill tumour cells through mechanisms at the molecular level^31^. NKG2D.ζ–NK cell, a gene-modified type of NK cell that targets myeloid-derived suppressor cells, enhances CAR-T cell activity in NB^32^. Similarly, increased uptake of cancer-derived neo-antigens by dendritic cells can stimulate the antitumor effect of CD8 + T cells, indicating the vital role of antigen processing in cancer immunity^33^. This study showed that the 21-gene model was highly involved in regulating the tumour immune microenvironment, and the findings of the afore-mentioned immunoassays reveal that the high-risk group in our model has a limited effect on immunotherapy.

Drug sensitivity analysis showed that the 21-gene model risk score was significantly correlated with patient sensitivity to bexarotene, camptothecin, docetaxel, metformin, mitomycin C, and viNBastine. Bexarotene, camptothecin, mitomycin C, and viNBastine are conventional chemotherapeutic drugs for NB treatment, and early studies have reported that the camptothecin analogue, gimatecan, is active in vitro in human NB^34^. Recently, it was found that the combination of nano-formulated docetaxel and curcumin in injectable nanoparticles significantly improved the efficacy in orthotopic models of NB^35^. In addition to being an anti-diabetic drug, metformin also has anti-proliferation and anti-growth properties in several tumours. In vitro cell experiments have shown that metformin may inhibit the growth and proliferation of NB SH-SY5Y cells through the Erk1/2 and Cdk5 pathways^36^. Since there are more than 10,000 small-molecular compounds in the early stages of drug discovery and development, evaluating the activity of all these small-molecular compounds is technically challenging, and the relevant procedures are expensive and time-consuming^37^. Wang et al.^37^ developed a novel deep learning predictive model, called DMFGAM, to predict hERG blockers. Validation experiments were conducted to evaluate the performance of DMFGAM. The results showed that DMFGAM is a useful tool for classifying small-molecule drugs into hERG blockers and hERG non-blockers. This tool can also be used to verify the effectiveness and related side effects of newly discovered small-molecule compounds for the treatment of NB.

By studying the specific signalling pathways involved in high-risk and low-risk models, I found that the GO-enriched pathways include CALCIUM DEPENDENT CELL–CELL ADHESION VIA PLASMA MEMBRANE CELL ADHESION MOLECULES and MITOCHONDRIAL RESPIRATORY CHAIN COMPLEX ASSEMBLY; KEGG-enriched pathways include DORSO-VENTRAL AXIS FORMATION and HUNTINGTON’S DISEASE, suggesting that the disturbance of these signalling pathways in the high-risk and low-risk groups affected prognosis in NB. Signal transduction networks are largely composed of proteins that can be modified or degraded, as well as interact with and move to specific cellular locations. Liquid–liquid phase separation (LLPS) is a crucial mechanism for regulating biological functions by controlling the spatiotemporal distribution of intracellular biomacromolecules^38^. To explore how different mRNAs compete to bind to the same protein partner in biological cells, Xu et al.^38^ proposed a Cahn-Hillard phase-field model paired with a Ginzburg–Landau free-energy scheme to describe high-valence mRNA-protein interactions to form distinct complexes capable of phase separation and perform different biological functions. They also found that the gradient-interfacial energy coefficients, initial mRNAs levels, and mRNA-protein binding rates could efficiently shift the spatial patterns of the two specific droplets from segregation to shared interface or enclosed patterns. This study sheds light on the molecular mechanisms underlying agglutinant assembly in LLPS and provides potential clues for the development of more rational disease treatment strategies. An advantage of ordinary differential equation (ODE) models is that they describe the rate of change of continuous variables used to model dynamic systems in several areas^39^. ODE modeling can be used to study the effects of multifunctional antitumor drugs on human tumor cells and normal cells and the specific signaling pathways involved, as well as to explore the conversion mechanism between various death modes (ferroptosis and apoptosis) in single cells, providing potential clues to guide the development of more rational control strategies for diseases^40^. Wang et al.^41^ established a model built using a GCN with a conditional random field, called GCNCRF, to predict potential relationships between lncRNA and miRNA. The GCNCRF utilizes an attention mechanism to update the node weights so that each node can reassign weights according to the difference between neighboring nodes. Compared to existing state-of-the-art methods, the proposed method achieves a higher predictive accuracy. We can also use GCNCRF to predict the potential relationships between miRNAs and mRNA in future biological information research.

Finally, I used the 21 model genes as the gene set for this analysis and found that they were regulated by multiple transcription factors and other common mechanisms. The analysis showed that the motif annotation with the highest normalized enrichment score (NES: 5.02) was cisbp__M2248. Five genes were enriched in this motif, which were (in order): AATF, FGL2, FOXO3, IFI6 and LEFTY1.Studies have shown that the apoptosis-antagonising transcription factor (AATF) acts through the p38/MK2/AATF signalling pathway as a critical repressor of p53-driven apoptosis in tumour cells, implicating this signalling cascade as a novel target for chemotherapy-sensitising therapeutic efforts^42^. FGL2, a member of the thrombospondin family, is essential to regulate the activity of immune and tumour cells in glioblastoma (GBM). FGL2 has immunosuppressive effects in the GBM tumour microenvironment and the ability to promote the progression of malignant tumours, making it a potential new target gene for GBM immunotherapy^43^. Previous studies have shown that FOXO3, a member of the fork-head box O (FOXO) family, regulates autophagy in various cells. FOXO3 inhibits human gastric adenocarcinoma cell growth by promoting autophagy in an acidic microenvironment^44^. In ovarian cancer (OC), it was found that interferon-α inducible protein 6 (IFI6) was found to promote the proliferation of OC cells by activating the NF-κB pathway and induces cisplatin resistance^45^. In addition, the left–right determination factor (LEFTY) is a novel member of the transforming growth factor-β superfamily. LEFTY expression has been recognised as a stemness marker because LEFTY is enriched both in undifferentiated embryonic stem cells and blastocysts^46^. The results of qRT-PCR showed that AATF, FGL2, FOXO3, and LEFTY1 were upregulated in the NB cell line, compared to the healthy liver cell line (QSG-7701).

## Conclusion

By integrating scRNA-seq and RNA-seq data, I performed multiple machine-learning methods and established a novel prognostic model for OS prediction in patients with NB that could be applied to predict the survival probability of these patients. Furthermore, the risk score is a promising independent prognostic factor closely correlated with the immune microenvironment and clinicopathological characteristics. Overall, this study provides information on reliable predictors of NB treatment efficacy, opening up new avenues for the targeted treatment of NB in the future.

## Materials and methods

### Data acquisition

The TCGA database (https://portal.gdc.cancer.gov/), the largest database of cancer gene information available, holds data including gene expression data, miRNA expression data, copy number variants, DNA methylation, single nucleotide polymorphisms, and more. We downloaded the original mRNA expression data from processed TARGET-NB for 160 samples. The Gene Expression Omnibus (GEO) database (https://www.ncbi.nlm.nih.gov/geo/info/datasets.html) is a gene expression database created and maintained by the US National Centre of Biotechnology Information (NCBI). We downloaded NB-related data, GSE192906, from the NCBI GEO public database for single-cell correlation analysis, totalling five samples. We also downloaded the series matrix file data for GSE62564 from the NCBI GEO public database, which was annotated on the platform GPL11154. Then, we extracted data for 498 patients with NB with complete expression profiles and survival information. Additionally, we downloaded the series matrix file data for GSE85047 from the NCBI GEO public database, which was annotated on the platform GPL5175. Then, we extracted data from 269 patients with NB with complete expression profiles and survival information.

### Single-cell analysis

The expression profile was first read through the Seurat package^47^ and screened for low expression genes (nFeature_RNA > 100 & percent.mt < 15). Data were sequentially normalised, homogenised, and subjected to principal component analysis (PCA). The optimal number of principal components (PCs) was determined using ElbowPlot(17), and t-SNE (t-distributed stochastic neighbour embedding) analysis^48^ was used to obtain the positional relationships between each cluster. Clusters were annotated using the BlueprintEncodeData annotation file provided by the Celldex package^49^ and assigned separately to cells with important relationships to the onset of the disease. Finally, we extracted the marker genes for each subtype of the cells from the single-cell expression profile by setting the logfc.threshold parameter of FindAllMarkers at 0.585 and the min.pct parameter at 0.25. Genes where p_val_adj was < 0.05 and |avg_log2FC| was > 0.585 were selected as marker genes specific for each subtype.

### Analysis of ligand-receptor interactions

CellPhoneDB (database version. 4.0)^50^ is a publicly available repository of selected receptors, ligands, and their interactions. Both ligands and receptors contain subunit structures that accurately represent heteromeric complexes. The CellPhoneDB ligand-receptor database integrates data from UniProt, Ensembl, PDB, IUPHAR, and others. It stores 1,885 protein interactions, allowing for a comprehensive and systematic analysis of intercellular communication molecules and the study of intercommunication between different cell types and communication networks. We performed a significant analysis of ligand-receptor relationships by calling the statistical analysis function of the software package CellphoneDB on the features in the single-cell expression profiles. We set the cluster labels of all cells to be randomly arranged 1,000 times. We determined the mean expression levels of receptors in the clusters and their interacting ligands in the interacting clusters. For each receptor-ligand pair in each comparison between the two cell types, this generated a null distribution (also known as the Bernoulli distribution or the binomial distribution). Finally, we selected some ligand-receptor pairs of interest for the presentation of relationship pairs.

### Functional enrichment analysis of the genes

Functional annotation of important gene sets was performed through the Metascape database (www.metascape.org) to thoroughly explore the functional relevance of the gene sets. Gene ontology (GO) analysis and Kyoto Encyclopaedia of Genes and Genomes (KEGG) pathway analysis^51–53^ were performed for specific genes. A minimum overlap of ≥ 3 & *p* ≤ 0.01 was considered statistically significant.

### Model construction and prognosis

Prognosis-associated genes were selected, and a prognostic model was further constructed using Lasso regression^54^. After incorporating the expression values for each specific gene, a risk score formula was constructed for each patient and weighted by their estimated regression coefficients in Lasso regression analysis. Patients were divided into low-risk and high-risk groups according to the risk score formula using the median risk score as the cut-off. The difference in survival between the two groups was assessed using Kaplan − Meier analysis and compared using log-rank statistics. The role of risk scores in the prediction of the patient’s prognosis was examined using Lasso regression analysis and stratified analysis. In addition, receiver operating characteristic (ROC) curves were used to investigate the accuracy of the model predictions.

### Drug sensitivity analysis

Based on the largest pharmacogenomics database (Genomics of Drug Sensitivity in Cancer (GDSC), https://www.cancerrxgene.org/), we used the R package ‘pRRophetic’^55^ to predict the chemotherapy sensitivity of each tumour sample. Regression was used to determine the IC50 estimates for each specific chemotherapy drug treatment, and the regression and prediction accuracy were tested with 10 cross-validations using the GDSC training set. All parameters were set at default values, including during the removal of batch effects by ‘combats’ and the average of duplicate gene expression.

### Immune cell infiltration analysis

RNA-seq data from different subgroups of patients with NB were analysed using the CIBERSORT algorithm^56^ to infer the relative proportions of the 22 immune infiltrating cell types. Pearson’s correlation analysis was performed on the level of gene expression, as well as on the immune cell content. A P-value of less than 0.05 was considered statistically significant.

### Gene set enrichment analysis

Gene set enrichment analysis (GSEA; http://www.broadinstitute.org/gsea)^57^ was performed on the expression profile of NB to identify genes differentially expressed between high- and low-risk groups. Gene sets were filtered using maximum and minimum gene sets of the size of 500 and 15 genes, respectively. After 1,000 permutations, an enriched gene set was obtained based on a P-value of less than 0.05 and a false discovery rate value of 0.25. Finally, the significantly enriched pathways from GO and KEGG enrichments^51–53^ were separately presented in a concentrated display.

### Regulatory network analysis of important genes

In this study, transcription factors were predicted using the R package ‘RcisTarget’^58^. All calculations performed using RcisTarget are based on motifs. The normalised enrichment score (NES) of a motif depends on the total number of motifs in the database. In addition to the motifs annotated by the source data, we inferred further annotation into a file based on motif similarities and gene sequences. The first step in estimating the overexpression of each motif in a gene set was to calculate the area under the curve (AUC) for each pair of motif-motif sets. This was based on the calculation of the recovery curve of the motif ranking by gene sets. The NES for each motif was calculated from the AUC distribution of all motifs in the gene set. We used rcistarget.hg19.motifdb.cisbpont.500 bp for the Gene-motif rankings database.

### Cell culture and quantitative real‑time PCR (qRT‑PCR)

The NB cell line SK-N-AS was obtained from Wuhan Pricella. The healthy human liver cell line QSG-7701 was obtained from Cellverse. Cells were kept in Dulbecco’s modified Eagle’s medium supplemented with 10% foetal bovine serum (Wisent, Ottawa, ON, Canada) and 1% penicillin in humid conditions at 37 °C with a 5% CO_2_ atmosphere. The RNA from the cell lines, QSG-7701 and SK-N-AS, was extracted using TRIzol reagent (Invitrogen), and the RevertAid First-Strand cDNA Synthesis Kit (Thermo Fisher Scientific, Inc.) was used to synthesise cDNA. qRT-PCR analysis was performed using SYBR Green (Takara). The primer sequences are summarised in Table 1.

**Table 1:** Primer sequences.

GAPDH_F	GTCTTCACCACCATGGAGAA
GAPDH_R	TAAGCAGTTGGTGGTGCAG
FGL2_F	GCTGTTCTTGCCACTTACGG
FGL2_R	GGGCAGACATCCTTTGCTCT
AATF_F	GCAAAGGCAGGAAACTTCGG
AATF_R	GTTCTGTCCTGGCATCATCA
IFI6_F	GGGTGGAGGCAGGTAAGAAA
IFI6_R	GATCGCAGACCAGCTCATCA
FOXO3_F	GTCCGCGATCCTGTACGTG
FOXO3_R	CGTCTTCATCGTCCTCCTCC
LEFTY1_F	CTGTGACCCTGAAGCACCAA
LEFTY1_R	ACTGTCGAGGCCCCAGAA

### Statistical analysis

Survival curves were generated using the Kaplan–Meier method and compared using the log-rank test. Multivariable analysis was performed using the Cox proportional hazards model. All analyses in this study were performed using R (version 4.0). All statistical tests were two-sided and *P* < 0.05 was considered statistically significant.

### Supplementary Information

Supplementary Information.

## Data Availability

Publicly available datasets were analyzed in this study. This data can be found here: GSE192906 (https://www.ncbi.nlm.nih.gov/geo/query/acc.cgi?acc=GSE192906) TARGET-NBL (https://portal.gdc.cancer.gov/). GSE62564 (https://www.ncbi.nlm.nih.gov/geo/query/acc.cgi?acc=GSE62564). GSE85047 (https://www.ncbi.nlm.nih.gov/geo/query/acc.cgi?acc=GSE85047).
